# The complete plastome sequences of *Mangifera indica* L. (Anacardiaceae)

**DOI:** 10.1080/23802359.2017.1390407

**Published:** 2017-10-14

**Authors:** Sangjin Jo, Hoe-Won Kim, Young-Kee Kim, Jung-Yeon Sohn, Se-Hwan Cheon, Ki-Joong Kim

**Affiliations:** Division of Life Sciences, Korea University, Seoul, Korea

**Keywords:** Plastome, large inversion, Anacardiaceae, *Mangifera indica*, mango

## Abstract

In this study, we determined the complete plastome sequence of *Mangifera indica* L. (Anacardiaceae) (NCBI acc. no. KX871231). The complete plastome is 157,780 bp in length, and consists of a large single copy of 86,673 bp and a small single copy of 18,349 bp, separated by two inverted repeats of 25,792 bp. The plastome contains 112 genes, of which 78 are protein-coding genes, 30 are tRNA genes, and four are rRNA genes. Sixteen genes contain one intron and two genes have two introns. The average A-T content of the plastome is 62.1%. The *M. indica* plastome has approximately 15 kb inversion between *trn*T-UGU and *trn*T-GGU. We identify a palindromic repeat of 18 bp (ATTCTTTTTTTTTTTTTT/AAAAAAAAAAAAAAGAAT) near the inversion breakpoints of *M. indica* plastome. Phylogenetic analysis revealed that *M. indica* is a sister group of *Rhus chinensis* with 100% bootstrap support. Anacardiaceae clade is a sister group of *Boswellia sacra* (Burseraceae) with 100% bootstrap support.

*Mangifera indica* L., commonly known as mango, is a widely cultivated tropical fruit that originates in northeastern India, Myanmar and Bangladesh (Kim 2011). It is one of the most widely cultivated tropical fruits. It belongs to the family Anacardiaceae in the Sapindales (APG IV 2016). The complete plastome sequences of Anacardiaceae are previously reported from *Pistacia*, *Rhus*, and *Spondias*, etc. (Lee et al. 2016). The complete plastome sequence of *M. indica* will aid us in developing molecular markers for the identification and improvement of cultivars of this economically important species.

The leaves of *M. indica* used in this study were collected from the Korea University greenhouse, where we grew the plants from seeds originally collected from Thailand. A voucher specimen was deposited in the Korea University Herbarium (KUS acc. no. 2014-0249). Fresh leaves were ground into powder in liquid nitrogen and total DNAs were extracted using the CTAB method (Doyle and Doyle 1987). The DNAs were further purified by the ultracentrifugation and dialysis (Palmer 1986). The genomic DNAs are deposited in the Plant DNA Bank in Korea (PDBK acc. no. 2014-0249). The complete plastome sequence was generated using an Illumina HiSeq 2000 system (Illumina, Inc., San Diego, CA). An average coverage of sequence was 2201 times of the *M. indica* plastome. Annotations were performed using the National Center for Biotechnology Information (NCBI) BLAST, and tRNAscan-SE programs (Lowe and Eddy 1997).

The gene order and structure of the *M. indica* plastome are similar to those of a typical angiosperm (Shinozaki et al. 1986; Kim and Lee 2004; Yi and Kim 2012) except a large inversion. The *M. indica* plastome shows an approximately 15 kb large inversion in LSC region. It is located between *trn*E-UUC and *trn*L-UAA. We identified a palindromic repeat of 18 bp (ATTCTTTTTTTTTTTTTT/AAAAAAAAAAAAAAGAAT) near the inversion breakpoints. The repeats may have a role to generate the inversion. However, neither the inversion nor the repeat were found from other available Anacardiaceae plastomes such as *Rhus* and *Spondias* (Lee et al. 2016). The partial plastome sequences of *M. indica* were previously reported by Azim et al. (2014). However, they did not identify the inversion and their sequence is unavailable from the NCBI database.

The complete plastome is 157,780 bp in length and consists of a LSC of 86,673 bp and a small single copy (SSC) of 18,349 bp, separated by two inverted repeats (IR) of 26,379 bp. The plastome comprises 112 unique genes (78 protein-coding genes, 30 tRNA genes, and four rRNA genes). The average A-T content of the plastome is 62.1%. The A-T contents in the LSC, SSC, and IR regions are 64.0%, 67.6%, and 57.0%, respectively. Sixteen genes contain intron and two genes, *ycf3* and *clpP*, have two introns. The *inf*A gene is pseudogene. A total of 57 simple sequence repeat (SSR) loci are scattered among the plastome. Among these, 44, 6, and 7 are mono-SSR, di-SSR, and tri-SSR loci, respectively. Some of these loci will be useful in identifying cultivars of *M. indica*.

To validate the phylogenetic relationships of *M. indica* among Anacardiaceae, we constructed a maximum likelihood (ML) tree. Phylogenetic analysis was performed on a data set that included the 78 protein-coding genes and four rRNA genes from 28 taxa using RAxML v. 7.7.1 (Stamatakis et al. 2008). The 82 gene sequences (75,831 bp) were aligned with MUSCLE in Geneious v. 6.1.8 (Biomatters Ltd.; Kearse et al. 2012). *M. indica* is nesting on Anacardiaceae clades (Figure 1). This formed one clade with *Rhus chinensis*. And this clade is a sister group of *Spondias* clade with 100% bootstrap support value. Phylogenetic analysis revealed that Anacardiaceae formed one clade with Burseraceae with a 100% bootstrap support value. Anacardiaceae and Burseraceae clade with other Sapindales clades formed monophyletic clade with 100% bootstrap support value.

**Figure 1.**
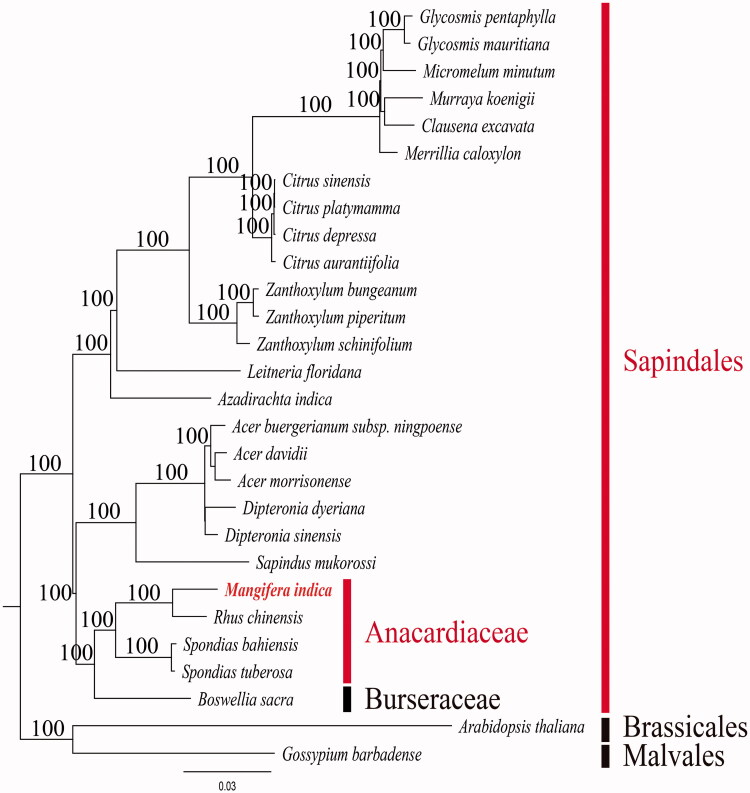
Maximum likelihood (ML) tree based on 78 protein-coding and four rRNA genes from 28 plastomes as determined by RAxML (–ln *L=* –281619.205218). The numbers at each node indicate the ML bootstrap values. Genbank accession numbers of taxa are shown below, *Acer buergerianum* subsp. *ningpoense* (KF753631), *Acer davidii* (NC_030331), *Acer morrisonense* (NC_029371), *Arabidopsis thaliana* (NC_000932), *Azadirachta indica* (NC_023792), *Boswellia sacra* (NC_029420), *Citrus aruntiifolia* (NC_024929), *Citrus depressa* (NC_031894), *Citrus playmamma* (NC_030194), *Citrus sinensis* (NC_008334), *Clausena excavata* (NC_032685), *Dipteronia dyeriana* (NC_031899), *Dipteronia sinensis* (NC_029338), *Glycosmis mauritiana* (NC_032686), *Glycosmis pentaphylla* (NC_032687), *Gossypium barbadense* (NC_008641), *Leitneria floridana* (NC_030482), *Mangifera indica* (KX871231**)**, *Merrilla caloxylon* (NC_032688), *Micromelum minutum* (NC_032689), *Murraya koenigii* (NC_032684), *Rhus chinensis* (NC_033535), *Sapindus mukorossi* (NC_025554), *Spondias bahiensis* (NC_030526), *Spondias tuberosa* (NC_030527), *Zanthoxylum bungeanum* (NC_023259), *Zanthoxylum piperitum* (NC_023259) and *Zanthoxylum schinifolium* (NC_023259).
